# Increased Sphingosine Kinase 1 Expression Is Associated with Poor Prognosis in Human Solid Tumors: A Meta-Analysis

**DOI:** 10.1155/2022/8443932

**Published:** 2022-01-28

**Authors:** Chuanmeng Zhang, Chenglin Zhou, Jie Xu, Shanshan Xue

**Affiliations:** ^1^The Center for Translational Medicine, Taizhou People's Hospital, Affiliated 5 to Nantong University, Taizhou, 225300 Jiangsu Province, China; ^2^Department of Clinical Laboratory, Taizhou People's Hospital, Affiliated 5 to Nantong University, Taizhou, 225300 Jiangsu Province, China

## Abstract

**Methods:**

PubMed, Web of Science, Embase, CNKI, and Wanfang databases were thoroughly searched for eligible studies, in which the relationship between SPHK1 expression and cancer prognosis was evaluated. Hazard ratios (HRs) and 95% confidence intervals (CIs) were pooled to estimate the impact of SPHK1 expression on cancer patients' survival. Odds ratios (ORs) and 95% CIs were combined to assess the association between SPHK1 expression and clinicopathological characteristics of cancer patients. The certainty of evidence was evaluated by Grading of Recommendations, Assessment, Development, and Evaluation (GRADE) criteria.

**Results:**

Thirty studies comprising 32 cohorts with 5965 patients were included in this meta-analysis. The outcomes indicated that elevated SPHK1 expression was associated with worse overall survival (OS) (HR = 1.71, 95% CI: 1.45-2.01, *P* < 0.001) and disease-free survival (DFS) (HR = 1.34, 95% CI: 1.13-1.59, *P* = 0.001). What is more, SPHK1 overexpression was significantly correlated with certain phenotypes of tumor aggressiveness, such as clinical stage (OR = 2.07, 95% CI: 1.39-3.09, *P* < 0.001), tumor invasion (OR = 2.16, 95% CI: 1.47-3.18, *P* < 0.001), lymph node metastasis (OR = 2.04, 95% CI: 1.71-2.44, *P* < 0.001), and distant metastasis (OR = 3.16, 95% CI: 2.44-4.09, *P* < 0.001). The quality of the evidence for both OS and DFS was low.

**Conclusions:**

Increased SPHK1 expression is related to poor prognosis in human cancers and may serve as a promising prognostic marker and therapeutic target for malignant patients. However, conclusions need to be treated with caution because of lack of high quality of evidence.

## 1. Introduction

Cancer is a serious public health problem, leading to a severe burden of disease in the world. According to a report released by GLOBOCAN in 2018, approximately 18.1 million people were newly diagnosed with cancer and 9.6 million people died of cancer, which was based on researches performed in 185 countries [1]. The mortality rate for all cancers combined continuously decreased 26% from 1991 to 2015, and the main reasons included improvements in cancer prevention, screening and early detection, and cancer treatment [2]. However, the 5-year overall survival rate is still low in the majority of cancer patients [3, 4]. Therefore, it is of great significance to find new biomarkers for the diagnosis and prognosis of cancers.

Sphingolipids are a key component of cancer development due to their role in angiogenesis and lymphangiogenesis driven by cancer stem cells [5]. Many researches have investigated the role of different sphingolipid enzymes, sphingolipid binding proteins, and transmembrane transporters in human cancer [6]. Among them, members of the sphingosine kinase (SPHK) family are key enzymes in cancer biology, because their catalytic activity is essential for regulating sphingolipid metabolism [5]. SPHK1, an isoenzyme of SPHK, catalyzes the phosphorylation of sphingosine to form sphingosine-1-phosphate (S1P), which inhibits cell apoptosis and promotes cell proliferation and angiogenesis [7, 8]. In contrast, sphingosine and ceramide, the metabolic precursors of S1P, stimulate apoptosis and inhibit cell proliferation [9]. Thus, the balance between these precursors and S1P within the cell has been proposed as a switch that drives the decision between cell proliferation and death [10]. In addition, the key regulator of this switch is SPHK1, which converts sphingosine into prosurvival S1P [11, 12].

SPHK1 has been shown to be significantly upregulated in a variety of cancers, such as breast cancer [5], lung cancer [13], head and neck carcinoma [14], and gastric cancer [15], which may be used as a procancer factor and therapeutic target and have an impact on diagnosis and treatment. Moreover, recent studies have demonstrated that high SPHK1 protein expression is associated with poor prognosis of many tumors [5, 15–35]. However, several publications showed that the association is nonsignificant [5, 16, 36–43]. Therefore, we conducted this meta-analysis to better assess the prognostic value of SPHK1 in tumors.

## 2. Materials and Methods

### 2.1. Literature Search

In accordance with the Preferred Reporting Items for Systematic Reviews and Meta-Analyses (PRISMA) statement, we performed a systematic literature search of PubMed, Web of Science, Embase, CNKI, and Wanfang databases to find relevant articles assessing the relationship between SPHK1 expression and prognosis of various malignant tumors. The following terms and their combinations are used as search keywords: (“sphingosine kinase 1” or “SPHK1”) AND (“immunohistochemistry” or “IHC”) AND (“cancer” or “tumor” or “neoplasms” or “carcinoma” or “malignancy”) AND (“prognosis” or “survival” or “outcome”), covering all articles published in English and Chinese as of July 2021. In addition, we manually screened the reference lists of the retrieved articles to seek for additional eligible studies.

### 2.2. Inclusion and Exclusion Criteria

Eligible articles in this meta-analysis were subject to the following inclusion criteria: (1) the publication investigated the relationship between SPHK1 expression and the prognosis (overall survival (OS) and disease-free survival (DFS)) of patients with solid tumors. (2) The expression of SPHK1 was detected in the primary cancer tissue by immunohistochemistry (IHC) stain and divided into “positive” and “negative” or “high” and “low” groups; and (3) hazard ratio (HR) and 95% confidence interval (CI) were readily available or could be calculated indirectly. Studies with any of the following flaws were excluded: (1) reviews, abstracts, letters, editorials, expert opinions, case reports, or animal experiments; (2) HR and 95% CI could not be obtained by sufficient information or data; and (3) studies with a sample size of less than 50.

### 2.3. Data Extraction and Quality Assessment

The data extraction in this meta-analysis was independently completed by two investigators (XSS and ZCM) independently using a standardized data-extract form and any divergence was adjudicated through discussion. The following data were collected: first author's name, publication year and language, study region, duration time, cancer type, sample size, follow-up time, detection method, cut-off value, number and proportion of patients with high SPHK1 expression, survival data, analysis method, and clinicopathological characteristics. If both univariate and multivariate analyses were used to calculate the HR for OS or DFS, the latter was preferred because the result was adjusted for confounding factors and was more accurate [44]. For the articles in which prognosis was plotted only as the Kaplan-Meier curves, the Engauge Digitizer V4.1 software was then applied to obtain survival data according to the method of Tierney et al. [45].

The quality of each included cohort study was independently assessed using the Newcastle-Quality Assessment Scale (NOS). According to the NOS scale, the quality of the cohort study is judged based on three contents: selection of the exposed and unexposed cohort (4 points), comparability of the two cohorts (2 points), and outcome assessment (3 points) [46]. Studies with a score ≥ 6 were considered as of high quality.

### 2.4. Quality of Evidence

The Grading of Recommendations Assessment, Development, and Evaluation (GRADE) method was applied to evaluate the quality of the evidence [47, 48]. The GRADE was based on the study design, risk of bias, inconsistency, indirectness, imprecision, and other considerations (including large effect, plausible confounders, and dose-response gradient) [49]. The quality of evidence was graded as high, moderate, low, or very low. The GRADE was assessed using the website http://gradepro.org.

### 2.5. Statistical Analysis

HRs and their 95% CIs were combined to estimate the effect of SPHK1 expression on survival. ORs and their 95% CIs were pooled to assess the association between SPHK1 expression and clinicopathological features. The heterogeneity was evaluated using Cochran's *Q* and *I*-squared statistical tests, in which *P* < 0.05 or *I*^2^ ≥ 50% was considered significant heterogeneity [50]. When significant heterogeneity existed, the random effects model was used for analysis, including subgroup analysis. Otherwise, the fixed effects model was applied. Subgroup analysis was performed to further evaluate the prognostic value of SPHK1. Sensitivity analysis was performed by omitting each cohort study in turn to assess the impact of single cohort on the combined results. Potential publication bias was quantitatively evaluated using Begg's and Egger's asymmetry tests and visually evaluated by funnel plots [51]. If publication bias was found, the trim and fill method was performed to validate the reliability of the meta-analysis results. All analyses were conducted using STATA version 12.0 software (Stata Corporation, College Station, TX), and *P* < 0.05 was considered statistically significant.

## 3. Results

### 3.1. Literature Search and Study Demographics

**Figure 1:**
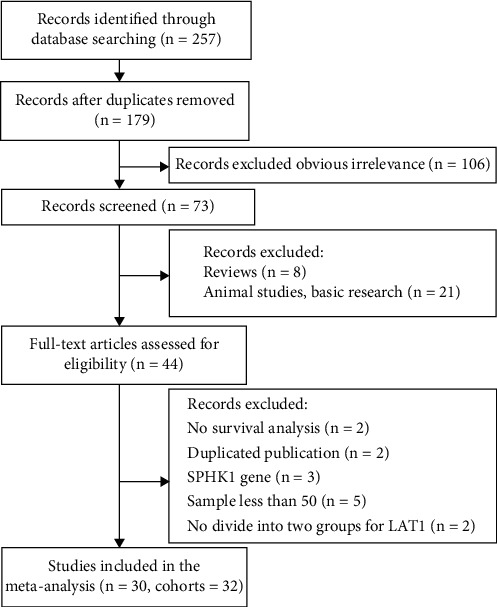
Flow diagram of the study selection process and specific reasons for exclusion in the meta-analysis.

The literature search flow diagram is presented in Figure 1. A total of 257 publications were initially retrieved from the PubMed, Web of Science, EMBASE, CNKI, and WanFang database. After removing duplicates and obviously irrelevant research, 73 articles were further screened. Then, 29 papers were excluded by screening the titles and abstracts. Of the remaining 44 potentially relevant articles, 14 studies were excluded because they did not fulfill the inclusion criteria or met one of the exclusion criteria. Finally, 30 studies with 32 cohorts were included in the meta-analysis.

The main characteristics of the 32 eligible cohorts are summarized in Table 1. Among the 32 cohorts, 5965 patients were included, with samples sizes that ranged from 51 to 1005. The publication years of the included cohorts ranged from 2008 to 2021. The large majority of cohorts were performed in Asia (twenty in China [15, 17–25, 27–33, 35, 41], two in Korea [5, 37], one in Japan [38], and one in Taiwan [26]), followed by Europe (two in Czech Republic [16], two in UK [34, 43], and one in Germany [42]), America (one in Canada [36] and one in USA [39]), and finally Australasia (one in Australia [40]). Among these included cohorts, 17 different cancer types were evaluated, including 5 breast cancer (BC) [24, 34, 40, 42, 43], 4 gastric cancer (GC) [15, 18, 32], 4 lung cancer (LC) [16, 29, 30], 3 colorectal cancer (CRC) [19, 20, 37, 39], 3 hepatocellular carcinoma (HCC) [22, 23, 28], and 1 each of ovarian carcinoma (OC) [36], papillary thyroid carcinoma (PTC) [17], renal cell carcinoma (RCC) [21], oral squamous cell carcinoma (OSCC) [38], pancreatic cancer (PC) [25], nasopharyngeal carcinoma (NPC) [27], cervical cancer (CC) [5], cholangiocarcinoma (CCA) [26], bladder cancer (BLC) [41], esophageal carcinoma (ESCC) [31], salivary gland carcinoma (SGC) [33], and astrocytomas (AC) [35]. The expression of SPHK1 was detected by IHC. 29 cohorts reported the correlation between SPHK1 expression and OS [5, 15, 16, 18–33, 35–42], while 11 cohorts evaluated the relationship between SPHK1 expression and DFS [5, 16, 17, 23, 24, 28, 34, 40, 42, 43]. According to the NOS score, each cohort included in this study gained a score of 6 or more, indicating that the articles were of high quality.

### 3.2. Association between SPHK1 Expression and Prognosis

**Figure 2:**
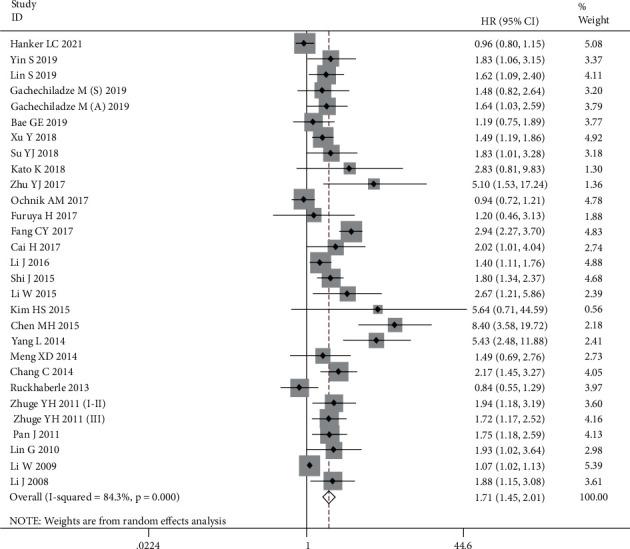
Forest plots of the overall outcomes for overall survival.

As shown in Table 2, a comprehensive analysis was conducted to assess the prognostic value of SPHK1 in human cancer. Twenty-nine cohorts comprising 5466 patients reported the association between SPHK1 expression and OS. The combined HRs indicated that high SPHK1 expression was obviously associated with poor OS (HR = 1.71, 95% CI: 1.45-2.01, *P* < 0.001) using the random-effects model because of heterogeneity (*I*^2^ = 84.3%, *P* < 0.001) (Figure 2). To further examine the prognostic value of SPHK1, subgroup analyses using random-effects model were performed by cancer type, sample size, proportion of patients with high SPHK1 expression, and analysis method. Subgroup analysis of cancer type showed that increased SPHK1 expression was significantly related to poor OS in patients with digestive system malignancies (HR = 1.79, 95% CI: 1.39-2.31, *P* < 0.001), urinary system cancers (HR = 1.49, 95% CI: 1.20-1.84, *P* < 0.001), head and neck cancers (HR = 2.08, 95% CI: 1.48-2.91, *P* < 0.001), and LC (HR = 2.15, 95% CI 1.39-3.35, *P* = 0.001), but no significant relationship was observed in patients with reproductive system tumors (HR = 1.71, 95% CI: 0.34-8.64, *P* = 0.519) and BC (HR = 1.16, 95% CI: 0.66-2.02, *P* = 0.608). In term of sample size, SPHK1 positive expression was significantly associated with poor OS in the subgroups with large (HR = 1.45, 95% CI: 1.17-1.80, *P* = 0.001) and small (HR = 1.97, 95% CI: 1.58-2.45, *P* < 0.001) sample sizes. With regard to the proportion of patients with high SPHK1 expression, high SPHK1 expression predicted shorter OS in both high (HR = 2.07, 95% CI: 1.55-2.75, *P* < 0.001) and low (HR = 1.44, 95% CI: 1.18-1.76, *P* < 0.001) proportion subgroups. Similarly, SPHK1 overexpression was associated with poor OS in the subgroup of analysis method. Thus, almost all subgroup analyses showed that SPHK1 positive expression was associated with poor OS, which to some extent indicates the prognostic value of SPHK1 for tumors and the stability of the results of this study.

**Figure 3:**
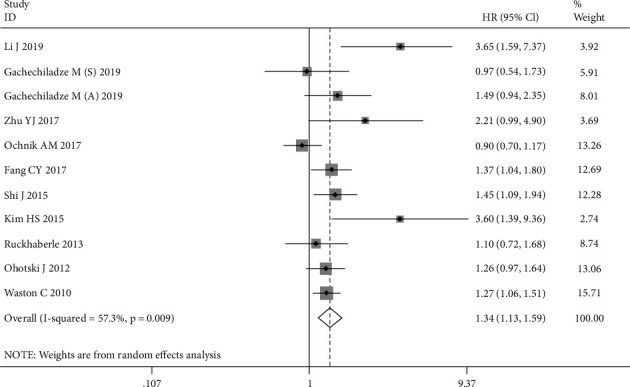
Forest plots of the overall outcomes for disease-free survival.

Eleven cohorts including 1839 cancer patients reported the impact of SPHK1 on DFS. Due to the obvious statistical heterogeneity (*I*^2^ = 57.3%, *P* = 0.009), the random model was applied and a significant association was observed between increased SPHK1 expression and poor DFS of cancer patients (HR = 1.34, 95% CI: 1.13-1.59, *P* = 0.001) (Figure 3).

### 3.3. Association between SPHK1 Expression and Clinicopathological Features

The relationship between SPHK1 expression and clinicopathological features is shown in Table 3. Seventeen cohorts with 3951 patients reported the association between SPHK1 expression and clinical stage, and the combined result indicated that high SPHK1 expression was obviously related to clinical stage (I-II vs. III-IV) (OR = 2.07, 95% CI: 1.39-3.09, *P* < 0.001). Similar results showed that high SPHK1 expression was significantly associated with tumor invasion (T1-T2 vs. T3-T4) (OR = 2.16, 95% CI: 1.47-3.18, *P* < 0.001), lymph node metastasis (negative vs. positive) (OR = 2.04, 95% CI: 1.71-2.44, *P* < 0.001), and distant metastasis (negative vs. positive) (OR = 3.16, 95% CI: 2.44-4.09, *P* < 0.001). This obvious association was not observed in age (young vs. old) (OR = 1.47, 95% CI: 0.80-2.70, *P* = 0.217), gender (male vs. female) (OR = 1.02, 95% CI: 0.87-1.19, *P* = 0.813), and tumor size (small vs. large) (OR = 1.36, 95% CI: 0.85-2.18, *P* = 0.199).

### 3.4. Sensitivity Analysis and Publication Bias

**Figure 4:**
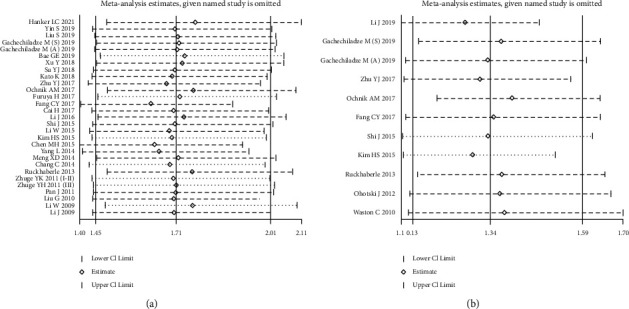
Effects of individual studies on pooled hazard ratios for SPHK1 expression and survival in solid tumors. (a) Result of sensitivity analysis for pooled overall survival estimation. (b) Result of sensitivity analysis for pooled disease-free survival estimation.

Sensitivity analysis was conducted to evaluate the influence of each cohort on the meta-analysis results of OS by omitting one cohort in turn. The results confirmed the robustness and reliability about the prognostic value of high SPHK1 expression on unfavorable OS (Figure 4(a)). Similarly, there was no significant change after omitting any cohort on the impact of SPHK1 expression on DFS, indicating the stability of our meta-analysis (Figure 4(b)).

**Figure 5:**
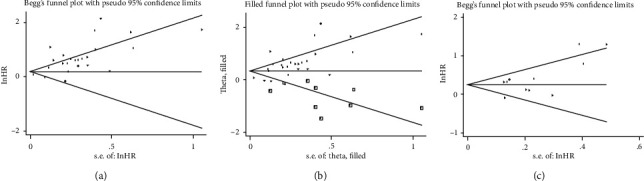
Begg's funnel plots for assessment of potential publication bias in studies of SPHK1 in patients with solid tumor. Each study is represented by one circle. The horizontal line represented the pooled effect estimate. (a) Funnel plot of publication bias for studies reporting overall survival. (b) Funnel plot adjusted with trim and fill methods for studies reporting overall survival. (c) Funnel plot of publication bias for studies reporting disease-free survival.

Both Begg's and Egger's tests were used to assess the potential publication bias of OS and DFS. For OS, the funnel plot showed a certain degree of asymmetry and was also confirmed by the Egger‘s test (*P* < 0.001), while the *P* value of the Bgger's test was greater than 0.05 (*P* = 0.399). Thus, the trim and fill method was used. Nine missing cohorts were needed to fill into the funnel plot (Figure 5(b)), and the adjusted HR (HR = 1.42, 95%CI = 1.21 − 1.65, *P* < 0.001) still showed the significant relationship between SPHK1 overexpression and worse OS, indicating the reliability of our results. In addition, there was no potential publication bias for DFS (Begg's test, *P* = 0.062; Egger' test, *P* = 0.082) (Figure 5(c)).

### 3.5. Level of Evidence

The GRADE method was adopted to assess the certainty of the evidence. The results indicated that the quality of the evidence for both OS and DFS was low (Table 4). Thus, our confidence in the effect estimate is limited: the true effect may be substantially different from the estimate of the effect.

## 4. Discussion

To date, some original articles have studied the prognostic significance of SPHK1 in solid tumors, and both significant and insignificant studies have emphasized the importance of SPHK1 for survival, so it is necessary to quantitatively summarize the survival results. The current meta-analysis included 32 cohorts with 5965 patients, and the systematically evaluated outcomes indicated that high SPHK1 expression was significantly associated with poor OS (HR = 1.71, 95% CI: 1.45-2.01, *P* < 0.001) and DFS (HR = 1.34, 95% CI: 1.13-1.59, *P* = 0.001) in various tumors. Moreover, sensitivity analysis and publication reinforced the stableness and reliability of the meta-analysis results. However, the quality of the evidence for both OS and DFS was low due to observational studies and heterogeneity. In addition, in order to further study the prognostic value of SPHK1 in solid tumors, we analyzed the relationship between SPHK1 and clinicopathological features that are also related to the prognosis of cancer patients. The pooled results demonstrated that SPHK1 overexpression was significantly related to clinical stage, tumor invasion, lymph node metastasis, and distant metastasis. Therefore, increased SPHK1 expression may be statistically associated with poor prognosis in cancer patients, although the certainty of evidence is low.

Studies have shown that the abnormal expression of SPHK1 can promote the occurrence of cancer and tumor progression. For example, SPHK1 regulates cell proliferation and apoptosis. SPHK1 can be activated by various growth factors, cytokines, and mitogens, such as vascular endothelial growth factor, platelet-derived growth factor, and tumor necrosis factor-*α* [52, 53]. The phosphorylation of SPHK1 and its subsequent translocation from the cytoplasm to the cell membrane were reported to be key factors in the acquisition of malignant phenotypes of cells through promoting the proliferation of malignant cells and protecting the apoptotic pathway from being destroyed [54]. Overexpression of SPHK1 promoted the proliferation in several tumor types, while blockade of SPHK1 inhibited tumor growth [55–58]. Moreover, changing the subcellular localization of SPHK1 had a significant impact on cell function, with cell membrane-translocated exhibiting an effective inhibitory effect on the G1-S phase transition of 3T3-L1 fibroblasts, indicating that the localization of SPHK1 in cells may play a crucial role in the apoptosis of tumor cells [17].

In addition, SPHK1 is also one of the important molecules involved in the invasion and metastasis of malignant tumors. Epithelial-mesenchymal transition (EMT) is originally a developmental procedure that has been used by tumor cells to promote their migration, invasion, and eventually colonization at a distance, resulting in distant metastasis, which leads to a poor prognosis of cancer patients [59]. Loss of E-cadherin and acquisition of vimentin are two critical steps in EMT [60]. Previous studies confirmed that overexpression of SPHK1 was associated with decreased expression of E-cadherin and increased expression of vimentin, suggesting that SPHK1 plays a pivotal role in the EMT of invasive carcinoma cells [19, 38, 61]. In addition, it was reported that SPHK1 promoted metastasis of cancer cells by inducing EMT, which was mediated by the FAK/AKT/MMPs axis [19]. Furthermore, it has been suggested that S1P released by SPHK1 activity promotes EMT in cancer by inhibiting the Snail-matrix metalloproteinases and remodeling the glycocalyx signaling pathway [62].

Thus, this study demonstrated that high immunohistochemical expression of SPHK1 in cancer tissues may be associated with poor survival in human solid tumor patients, which will promote the development of a novel biomarker for the diagnosis and prognosis and targeted therapy of solid tumors. First of all, in addition to biopsy or surgical tissue, further research on whether this important marker is also detectable in other forms of patient samples, such as blood, will be very important for the early detection and diagnosis of tumors [15]. Second, SPHK1 expression can be used as a reliable biomarker to grade the prognosis of cancer patients [37]. Finally, targeting SPHK1 or its downstream targets with clinically available inhibitors would be effective for tumor therapy, such as increasing tumor sensitivity to chemotherapy [36, 63, 64]. Therefore, further preclinical and clinical development of SPHK1 inhibitors is necessary for the treatment of tumors [5].

Apart from the inspiring results, several limitations still should be noted in this quantitative meta-analysis. First, the population of the included studies was mainly concentrated in Asia, which affected the applicability of the results to some extent. Second, the inconsistent cut-off values for distinguishing between high and low expression of SPHK1 and the different analysis methods for evaluating the correlation between SPHK1 overexpression and prognosis may lead to different results of the included studies. Third, all of the included studies were retrospective cohort studies, in which positive results are more likely to be published than negative results. Fourth, as some studies did not directly provide the HRs, we had to estimate the HRs and 95% CIs from the survival curves, which may cause some errors. Fifth, the publication bias of this study is a concern because articles with positive results are more likely to be published, which may exaggerate the connection between SPHK1 expression and adverse outcomes. Sixth, the quality of the evidence for both OS and DFS was low, which affected our confidence in the estimation of the effect. Finally, this meta-analysis was not prospectively registered in international prospective register of systematic reviews (PROSPERO) that can help reduce selective reporting of outcomes [65].

## 5. Conclusion

In summary, high SPHK1 expression was associated with poor prognosis and served as a useful prognostic biomarker, which might be a promising therapeutic target for solid tumors. However, conclusions need to be treated with caution because of lack of high quality of evidence.

## Figures and Tables

**Table 1:** Main characteristics of the eligible studies.

Study	Region	Duration	Cancer type	Sample size	Clinical stage	Follow up (months)	Detection method	Cut-off value	SPHK1-high (%)	Survival analysis	Language	Quality
Hanker 2021	Canada	1984-2009	OC	1005	I-IV	NR	IHC	≥3	248 (24.7)	OS (M)	English	7
Yin 2019	China	2003-2010	GC	120	I-III	NR	IHC	≥6	44 (36.7)	OS (M)	English	7
Li 2019	China	NR	PTC	92	I-IV	≥120	IHC	≥4	35 (38.0)	DFS (M)	English	8
Liu 2019	China	2013	CRC	114	I-IV	NR	IHC	≥3	78 (68.4)	OS (U)	English	6
Gachechiladze (S) 2019	Czech Republic	1996-2000 2005-2011	NSCLC	51	I-IV	NR	IHC	Median	34 (66.7)	OS (U), DFS (U)	English	6
Gachechiladze (C) 2019	Czech Republic	1996-2000 2005-2011	NSCLC	81	I-IV	NR	IHC	Median	28 (34.6)	OS (U), DFS (U)	English	6
Bae 2019	Korea	NR	CRC	328	I-III	NR	IHC	≥4	112 (34.1)	OS (M)	English	7
Xu 2018	China	NR	RCC	358	I-IV	60	IHC	﹥6	243 (67.9)	OS (U)	English	6
Su 2018	China	2013	CRC	92	NR	Median 54	IHC	≥10%	78 (84.8)	OS (U)	English	6
Kato 2018	Japan	1996-2014	OSCC	69	I-IV	60	IHC	≥6	38 (55.1)	OS (M)	English	8
Zhu 2017	China	2006-2013	BC	122	I-III	Median 56.5	IHC	≥8	64 (52.5)	OS (M), DFS (M)	English	8
Ochnik 2017	Australian	NR	BC	236	I-III	Median 61	IHC	≥2	123 (52.1)	OS (M), DFS (M)	English	7
Furuya 2017	USA	2008	CRC	85	I-IV	Median 57	IHC	≥50%	9 (10.6)	OS (M)	English	7
Fang 2017	China	2008-2011	HCC	252	I-IV	NR	IHC	≥40%	181 (71.8)	OS (U), DFS (U)	Chinese	6
Cai 2017	China	2008-2010	HCC	127	I-IV	60	IHC	NR	93 (73.2)	OS (M)	English	8
Li 2016	China	NR	PC	388	I-IV	60	IHC	≥6	230 (59.3)	OS (M)	English	8
Shi 2015	China	2000-2004	HCC	199	I-IV	36	IHC	≥20%	136 (68.3)	OS (U), DFS (U)	English	6
Li 2015	China	2007-2009	NPC	142	I-IV	60	IHC	≥6	93 (65.5)	OS (M)	English	7
Kin 2015	Korea	2002-2009	CC	287	I-II	NR	IHC	≥4	183 (63.8)	OS (M), DFS (M)	English	7
Chen 2015	Taiwan	1986-2006	CCA	96	NR	60	IHC	≥50	64 (66.7)	OS (M)	English	8
Yang 2014	China	2007-2013	SCLC	76	NR	Median 24	IHC	NR	53 (69.7)	OS (M)	Chinese	7
Meng 2014	China	2005-2008	BLC	153	I-III	60	IHC	Median	82 (53.6)	OS (M)	English	7
Chang 2014	China	2009	NSCLC	93	I-III	Until 2018	IHC	≥4	48 (51.6)	OS (U)	Chinese	6
Ruckhäberle 2013	Germany	1999-2001	BC	112	I-III	Median 57.6	IHC	UQ	29 (25.9)	OS (U), DFS (U)	English	6
Ohotski 2012	UK	1995-1998	BC	140	NR	NR	IHC	NR	110 (78.6)	DFS (U)	English	6
Zhuge 2011 (I-II)	China	2001-2005	GC	63	I-II	60	IHC	≥6	28 (44.4)	OS (U)	Chinese	6
Zhuge 2011 (III)	China	2001-2005	GC	116	III	60	IHC	≥6	77 (66.4)	OS (U)	Chinese	6
Pan 2011	China	2001-2005	ESCC	124	NR	96	IHC	≥2	89 (71.8)	OS (U)	English	6
Waston 2010	UK	1980-1999	BC	267	NR	Mean 95	IHC	NR	95 (35.6)	DFS (U)	English	6
Liu 2010	China	1995-2004	SGC	159	I-IV	60	IHC	≥6	85 (53.5)	OS (M)	English	7
Li 2009	China	1997-2001	GC	175	I-IV	60	IHC	≥6	115 (65.7)	OS (M)	English	8
Li 2008	China	2000-2005	AC	243	I-IV	NR	IHC	≥6	100 (41.2)	OS (M)	English	8

OC: ovarian carcinoma; GC: gastric cancer; PTC: papillary thyroid carcinoma; CRC: colorectal cancer; NSCLC: nonsmall cell lung cancer; RCC: renal cell carcinoma; OSCC: oral squamous cell carcinoma; BC: breast cancer; HCC: hepatocellular carcinoma; PC: pancreatic cancer; NPC: nasopharyngeal carcinoma; CC: cervical cancer; CCA: cholangiocarcinoma; SCLC: small cell lung cancer; BLC: bladder cancer; ESCC: esophageal carcinoma; SGC: salivary gland carcinoma; AC: astrocytomas; S: surgery; C: chemotherapy; UQ: upper quartile; IHC: immunohistochemistry; OS overall survival; DFS: disease-free survival; M: multivariate analysis; U: univariate analysis; NR: none reported.

**Table 2:** Summary of the meta-analysis results.

Categories	Cohorts (patients)	HR (95% CI)	*I* ^2^ (%)	*P*_*h*_	*Z*	*P*
OS (all)	29 (5466)	1.71 (1.45-2.01)	84.3	<0.001	6.45	<0.001
Cancer type						
Digestive system	14 (2279)	1.79 (1.39-2.31)	89.0	<0.001	4.50	<0.001
Urinary system	2 (511)	1.49 (1.20-1.84)	0.0	0.998	3.68	<0.001
Reproductive system	2 (1292)	1.71 (0.34-8.64)	64.2	0.095	0.64	0.519
HNC	4 (613)	2.08 (1.48-2.91)	0.0	0.841	4.26	<0.001
LC	4 (301)	2.15 (1.39-3.35)	63.4	0.042	3.41	0.001
BC	3 (470)	1.16 (0.66-2.02)	74.0	0.021	0.51	0.608
Sample size						
≥150	12 (3783)	1.45 (1.17-1.80)	88.6	<0.001	3.42	0.001
<150	17 (1683)	1.97 (1.58-2.45)	59.5	0.001	6.07	<0.001
SPHK1-high						
≥60%	14 (2209)	2.07 (1.55-2.75)	90.4	<0.001	4.99	<0.001
<60%	15 (3257)	1.44 (1.18-1.76)	66.7	<0.001	3.64	<0.001
Analysis method						
Multivariate	17 (3811)	1.65 (1.34-2.04)	79.1	<0.001	4.73	<0.001
Univariate	12 (1655)	1.73 (1.43-2.08)	66.3	0.001	5.75	<0.001
DFS (all)	11 (1839)	1.34 (1.13-1.59)	57.3	0.009	3.36	0.001

All pooled HRs were calculated from random-effect model. HNC: head and neck cancer; LC: lung cancer; BC: breast cancer; OS overall survival; DFS: disease-free survival; HR: hazard ratio; CI: confidence interval; *P*: *P* value for statistical significance based on *Z* test; *P*_*h*_: *P* value for heterogeneity based on *Q* test.

**Table 3:** Meta-analysis of SPHK1 and clinicopathological features in cancer patients.

Categories	Cohorts (patients)	OR (95% CI)	*I* ^2^(%)	*P*_*h*_	*Z*	*P*
Age (young vs. old)	21 (4607)	1.47 (0.80-2.70)	94.9	<0.001	1.23	0.217
Gender (male vs. female)	20 (3515)	1.02 (0.87-1.19)^F^	15.9	0.256	0.24	0.813
Clinical stage (I-II vs. III-IV)	17 (3951)	2.07 (1.39-3.09)	79.6	<0.001	3.56	<0.001
Tumor invasion (T1-T2 vs. T3-T4)	10 (2062)	2.16 (1.47-3.18)	52.8	0.025	3.91	<0.001
Lymph node metastasis (negative vs. positive)	17 (2922)	2.04 (1.71-2.44)^F^	33.3	0.090	7.88	<0.001
Distant metastasis (negative vs. positive)	12 (2261)	3.16 (2.44-4.09)^F^	0.0	0.632	8.72	<0.001
Tumor size (small vs. large)	14 (2655)	1.36 (0.85-2.18)	84.8	<0.001	1.28	0.199

All pooled ORs were calculated from random-effect model except for cells marked with (fixed^F^). *P*_*h*_ denotes *P* value for heterogeneity based on *Q* test; *P* denotes *P* value for statistical significance based on *Z* test. OR: odds ratio; CI: confidence interval.

**Table 4:** Risk of bias assessment using the GRADE instrument.

Quality assessment							Quality	Importance
No. of studies	Study design	Risk of bias	Inconsistency	Indirectness	Imprecision	Other considerations		
OS								
29	Observational studies	Not serious	Serious	Not serious	Not serious	Publication bias strongly suspected; strong association; all plausible residual confounding would suggest spurious effect, while no effect was observed	⨁⨁◯◯ Low	Our confidence in the effect estimate is limited

DFS								
11	Observational studies	Serious	Serious	Not serious	Not serious	Strong association; all plausible residual confounding would suggest spurious effect, while no effect was observed	⨁⨁◯◯ Low	Our confidence in the effect estimate is limited

Question: “Is Upregulation of SPHK1 associated with poor prognosis in human solid tumors?”.

## Data Availability

The data supporting this meta-analysis are from previously reported studies and datasets, which have been cited. The processed data are available within the article.
